# Effects of the type and direction of support surface perturbation on postural responses

**DOI:** 10.1186/1743-0003-11-50

**Published:** 2014-04-07

**Authors:** Chiung-Ling Chen, Shu-Zon Lou, Hong-Wen Wu, Shyi-Kuen Wu, Kwok-Tak Yeung, Fong-Chin Su

**Affiliations:** 1 School of Occupational Therapy, Chung Shan Medical University, Taichung, Taiwan; 2 Occupational Therapy Room, Chung Shan Medical University Hospital, Taichung, Taiwan; 3 Department and Graduate School of Physical Education, National Taiwan Sport University, Taichung, Taiwan; 4 Department of Physical Therapy, Hung Kuang University, Taichung County, Taiwan; 5 Institute of Biomedical Engineering, National Cheng Kung University, 1 University Rd., Tainan 701, Taiwan; 6 Medical Device Innovation Center, National Cheng Kung University, Tainan, Taiwan

**Keywords:** Surface perturbation, Posture, EMG, Center of mass, Kinematics

## Abstract

**Background:**

Postural control is organized around a task goal. The two most frequently used types of tasks for postural control research are translational (translation along the anterior-posterior axis) and rotational (rotation in sagittal plane) surface perturbations. These types of perturbations rotate the ankle joint, causing different magnitudes and directions of body sway. The purpose of this study was to investigate the effects of the type (translation vs. rotation) and direction (forward/toe up vs. backward/toe down) of the perturbation on postural responses.

**Method:**

Nineteen healthy subjects were tested with four perturbations, i.e., forward and backward translation and toe up and toe down rotation. The onset latency and magnitude of muscle activations, angular changes, and COM displacements were measured. In addition, the kinematic data were divided into two phases. The initial phase reflected the balance disturbance induced by the platform movement, and the reversal phase reflected the balance reaction.

**Results:**

The results showed that, in the initial phase, rotational perturbation induced earlier ankle movement and faster and larger vertical COM displacement, while translational and forward/toe up perturbations induced larger head and trunk angular change and faster and larger horizontal COM displacement. In the reversal phase, balance reaction was attained by multi-joint movements. Translational and forward/toe up perturbations that induced larger upper body instability evoked faster muscle activation as well as faster and larger hip or knee joint movements.

**Conclusions:**

These findings provide insights into an appropriate support surface perturbation for the evaluation and training of balance.

## Background

Postural perturbation is a sudden exposure to conditions that displace the body away from equilibrium. Externally induced postural perturbations are often applied in research to study feedback postural responses. The most common form of externally induced postural perturbation is support surface perturbation induced by a platform, which moves the base of support (BOS) under the body’s center of mass (COM) [1-7]. These support surface perturbations replicate the conditions of slipping, tripping, stepping on an irregular surface, or accelerating or decelerating the support surface during vehicular motion. According to the systems theory, postural control requires highly complex interactions among the sensory, motor and nervous systems. What’s more, postural responses are closely related to environmental constraints and task demands [8], such that the relative weights given to sensory inputs and the selection of postural strategy depend on the goal of the postural task. Platform rotation in sagittal plane and translation along the anterior-posterior axis are two biomechanically different postural tasks frequently used as support surface perturbation to test postural responses. Each type of perturbation induces very distinct postural responses in spite of the similarities in ankle joint rotation.

Early researchers used electromyography (EMG) to characterize patterns of muscle activity as a body responded to support surface perturbation. Nashner was first to observe that the subject’s postural responses to surface translation showed a stereotypical pattern with fixed muscle synergies [9,10]. In a later study, Horak and Nashner described two postural responses, an ankle strategy and a hip strategy, which could be used to maintain balance in response to surface translations [11]. Keshner et al. proposed that the activation patterns in response to forward and backward translations demonstrated temporal differences and that there were different patterns of muscle responses between rotational and translational perturbations [12]. Nardone et al. found that the antagonist reactions were induced only by rotational perturbations and suggested that late responses in the antagonist muscle were more closely connected with the type of postural imbalance than with the initial stretch of the leg muscles induced by the platform [13,14]. After the aforementioned studies, researchers combined EMG and videotaped recordings or a motion analysis system to examine postural responses that provided additional information about body kinematics. Differences in the kinematics of movement have been observed based on whether the platform displacements were anterior or posterior [15]. Two components of movement, i.e., an early passive component and a later active component, were identified in body kinematics. The early passive component was induced by the platform movement, and the later active component was a corrective response to the platform movement [16,17]. Szturm and Fallang further defined the components as two phases, i.e., a balance disturbance phase and a balance reaction phase. The results of their study showed that the different types of platform displacements resulted in a distinct pattern of proprioceptive and vestibular signals that would convey information related to the magnitude of the balance disturbance. Also, they proposed that the magnitudes of balance reaction, i.e., peak hip, knee, and ankle angular displacements and magnitude of muscle responses, were scaled to the velocity and acceleration of the platform [18].

During platform translation, the body′s base of support moves to a new position relative to space, while, during rotation, the tilted support base remains stationary relative to space. Balance disturbance induced by platform translation would be greater than that induced by platform rotation. The balance reaction to platform translation is to move the COM forward or backward to the new position within the displaced BOS. For platform rotation, the balance reaction is to minimize the displacement of the COM and to restore postural stability on the tilted support base [18]. Rotational and translational perturbations all rotate the ankle joint, but they induce different magnitudes and directions of body sway, i.e., backward translation and toe down rotation induce anterior body sway, while forward translation and toe up rotation induce posterior body sway. During upright stance, the position of the center of foot pressure is slightly anterior to the lateral malleolus, i.e., it corresponds to 24 ± 11% of the BOS starting with 0% at the edge of the heel and 100% at the edge of the toe [19]. Posterior body sway potentially causes more instability for people who are standing than anterior body sway because of the limited posterior base of support. In addition to the biomechanical difference between forward and backward perturbation, Nonnekes et al. proposed that backward and forward perturbations may be processed by different neural circuits [20].

Although studies have compared muscle responses between platform rotation and translation and compared muscle responses and movement reaction between anterior and posterior translation, no EMG and kinematic data have been collected to test the effects of all four types of platform displacements, i.e., forward translation (FT), backward translation (BT), toe-up rotation (UR) and toe-down rotation (DR)—performed at the same velocity and on the same subjects. Thus, it remains unclear whether there are fundamental differences in postural responses between the two types and the two directions of perturbations. An improved understanding of the impact of perturbation on posture (balance disturbance) and postural responses (balance reaction) to different types of surface perturbation will increase knowledge about appropriate evaluation methods and training program for balance disorders.

Therefore, we measured the onset latency and magnitude of muscle activation, angular changes, and COM displacements to determine the effect of type and direction on postural responses. Two phases of angular changes and COM displacements were quantified. We speculated that translational perturbation or forward/toe up perturbation would induce greater balance disturbance and elicit earlier and larger muscle activation and angular changes in joints than would rotational or backward/toe down perturbation. In addition, our expectation was that the effect of type and direction would also be noted in COM displacements, such that translational or forward/toe up perturbations would induce and recover earlier and larger horizontal displacement and rotational perturbation would induce and recover earlier and larger vertical displacement.

## Methods

### Participants

Nineteen adults (12 males and 7 females), having a mean age of 21.8 (±1.6) years, participated in the study. Their average weight was 60.6 (±9.5) kg, and their average height was 168.4 (±8.8) cm. Participants had no history of neurological diseases or musculoskeletal injuries that could interfere with their balance. Anthropometric measurements were made according to anatomical landmarks. Before the experiment, all participants provided informed consent, and the protocol was approved by the Institutional Review Board.

### Instrumentation

A custom, computer-controlled, servomotor-driven, moveable platform provided forward/backward translational and toe up/toe down rotational perturbations [21]. The velocities of the platform movement were set at 500 mm/s for translations and 50 degree/s for rotations with a ramp onset and ramp offset acceleration/deceleration profile [22]. The amplitudes of the platform movement for translations and rotations were set at 70 mm and 7 degrees, respectively. These parameters allowed the production of similar ranges and rates of rotation about the ankle joint during translation and rotation [14].

The MA-300 system (Motion Lab Systems, Inc.) was used to collect EMG data. EMG recordings were obtained with surface preamplified electrodes placed over the muscle belly longitudinal to the predicted path of the muscle fibers. The tested muscles, which were on the right side, were the cervical paraspinae (NK EXT), neck flexor (NK FLX), thoracic paraspinae (T EXT), pectoralis major (PEC), lumbar paraspinae (L EXT) (segmental level L2-3), abdominal rectus (ABD R) (lateral to the umbilicus), biceps femoris (BF), rectus femoris (RF), medial gastrocnemius (MG) and tibialis anterior (TA). EMG signals were sampled at 1000 Hz, sampling occurred one second prior to perturbation, and the period of acquisition lasted three seconds and was synchronized with the motion analysis system.

Kinematic data were obtained from a six-camera EvaRT 4.2 motion analysis system (Motion Analysis Corp, Santa Rosta, CA, USA). Forty-one retro-reflective spherical markers were placed on selected bony landmarks of the subject [23]. Three additional markers were placed on the surface of the platform to define the global coordinate system. The origin of the global coordinate system was defined as the center of the platform’s surface. Data were sampled at 200 Hz and stored for post-processing.

### Experimental procedures

The participants stood barefooted on the platform with the mid-lines of the feet 12 cm apart and parallel to the sagittal plane. Marking tape was placed on the platform to ensure that foot placement remained consistent from trial to trial. The axis of the ankle joint was located above the support surface (0.039* body height [24]) parallel to the rotation axis of the platform, which was located 35 mm below the support’s surface. Therefore, the distance between the two axes was about 100 mm. The participants were instructed to stand upright with their arms hanging freely at their sides, their knees and hips fully extended, and their heads held erect to look directly forward at a mark on the wall at a distance of 2 m. The subjects were fitted with a loosely adjusted ceiling-suspended harness that did not restrict body segment motion. All participants were tested under four perturbation conditions, i.e., FT, BT, UR, and DR, with six trials for each perturbation. The perturbations were delivered in a random sequence and began one second after data collection was initiated. The first three recordings at each condition were discarded to reduce data variability for taking account of habituation effects [25]. The subjects were instructed to respond to the disturbance without moving their feet, and no pre-test information regarding perturbation type or sequence was provided. Occasional steps did occur during the experiment, in which case additional trials were conducted, and subsequent analyses were confined to the trials without steps.

### Data analysis

EMG signals were pre-amplified, full-wave rectified, and Butterworth band-pass filtered (10–480 Hz) to remove motion artifacts and environment noise. Then, render an envelope with an integrator, corresponding to a second order Butterworth low pass filter with a cut-off frequency 6 Hz, to represent a meaningful profile of muscle activity. The latency of the muscle responses was measured as the time interval between the onset of the platform movement and the beginning of the burst of muscle activity, which was defined as the time the activation first exceeded the baseline level plus two standard deviations (where the baseline is defined as the average activation level for the 100 ms before platform movement). The magnitude of muscle activity was measured by integrated EMG extending 200 ms from the onset of the burst of muscle activity. EMG amplitude was normalized by dividing the maximum amplitude recorded during maximum voluntary isometric contraction. The maximal voluntary contractions were tested by the standard procedure of manual muscle testing according to the technique of Hislop and Montgomery [26]. An experimenter provided a matching resistance to the participants during the maximal exertions for restraining the subject’s movement.

The trajectory data of all markers were smoothed using a generalized cross-validation spline smoothing (GCVSPL) routine at a cut-off frequency of 6 Hz [27]. The whole body’s COM was calculated using a weighted sum average of a 13-segment, biomechanical model and using the position data of the reflective markers based on the global reference coordinate system. Definitions of the joint coordinate system were according to the International Society of Biomechanics (ISB) recommendation. Three markers attached to the skin overlying the apex of the right temporal bone, and the right and the left temporomandibular joint were used to define the head coordinate system The markers attached on the processus xiphoideus, the sternal notch and the spinous process of the 7th cervical vertebra were used to define the trunk coordinate system. The Euler angle system was used to describe the orientation of a distal segment coordinate system relative to a proximal segment coordinate system. The first rotation about the y axis represented the flexion/extension angle, and the second rotation about the x' axis represented the adduction/abduction or side-bending angle. The third rotation about the z" axis represented segmental axial rotation [28-30].

The timing and extent of kinematic measures were analyzed by quantifying the onset latency, the magnitude of angular changes, and the COM displacements by visual inspection and by the use of a computer algorithm. Two phases of the angular and COM displacement could be identified, i.e., the initial movement phase and the reversal movement phase. The initial movement phase, reflecting the mechanical effects of sudden platform movement, which was passive in nature, and the reversal movement phase, beginning at the initial peak and in the opposite direction to the initial phase, reflecting the outcome of balance correction, which was an active phase. The onset latency of the initial movement was determined as the time from the onset of the platform movement to the time at which the first inflection of the baseline angular and COM displacement tracing occurred with a sustained change in the slope of the displacement tracing. Following the time to initial response, we also identified the time to first peak displacement (or the onset of the reversal movement) at which the displacement tracing first reversed direction with a sustained slope [16]. The magnitude of the angular and COM displacements of the initial phase was defined as the difference in the magnitude at the onset of initial displacement and the magnitude at the time of the first peak displacement. The magnitude of the second phase (reversal movement) was defined as the difference in magnitude of the displacement at the time of the first reversal and the magnitude at the time of the second reversal, or the returned baseline [18].

A series of two-way analyses of variance (ANOVA) with repeated measures was performed to determine the differences between the two perturbation types (translational vs. rotational) and between the two directions (forward/toe up vs. backward/toe down). The dependent variables were onset latency and magnitude of muscle activation, joint angular changes, and COM displacements. For multiple tests, the significance level was set at 0.01.

## Results

### EMG

**Figure 1:**
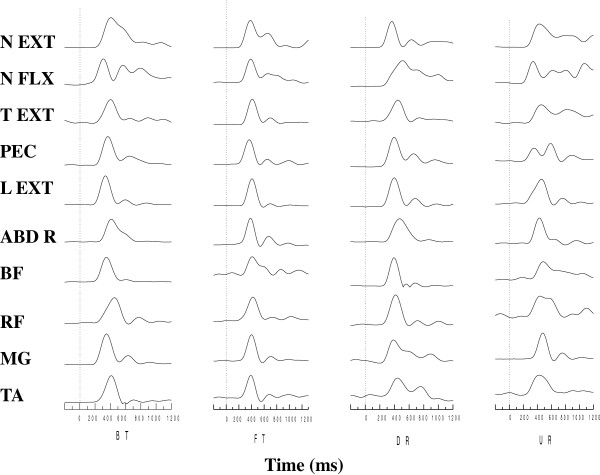
**EMG responses in four types of perturbation.** Averaged EMG responses of three trials of ten muscles for one representative subject in response to four types of perturbation. The vertical dotted line indicates the beginning of platform movement. NK EXT, cervical paraspinae; NK FLX, neck flexor; T EXT, thoracic paraspinae; PEC, pectoralis major; L EXT, lumbar paraspinae; ABD R, abdominal rectus; BF, biceps femoris; RF, rectus femoris; MG, medial gastrocnemius; TA, tibialis anterior; BT, backward translation, FT, forward translation, DR, toe down rotation; UR, toe up rotation.

**Figure 2:**
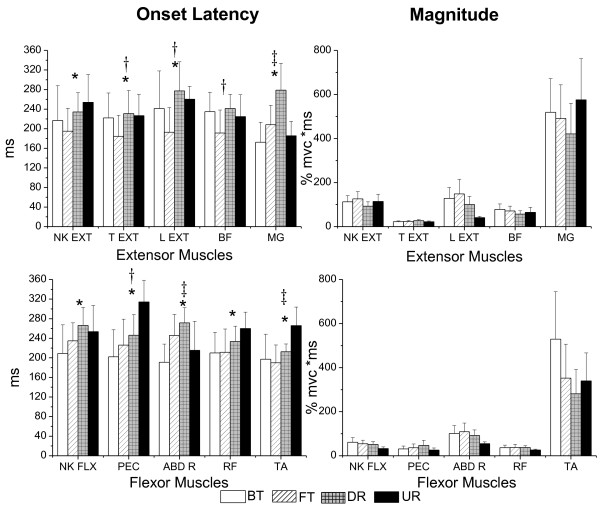
**Group means and standard deviation of the EMG data.** Group means and standard deviation of the EMG onset latency (left) and magnitude (right) for the extensor (top) and flexor (bottom) muscles for four types of perturbation. A significant difference between translation and rotation is indicated by*, a significant difference between forward/toe up and backward/toe down is indicated by†, and a significant interaction is indicated by‡.

The EMG records of ten muscles for one representative subject in response to four types of perturbation are presented in Figure 1. Mean onset of all the muscles were within 250 ms. The group means and standard deviation of onset latency and magnitude in each muscle for the four types of perturbation are shown in Figure 2. A significant difference between translational and rotational perturbation in the effect on onset latency was seen in all the muscles (F _(1, 18)_ = 8.36 to 14.51 for extensor muscles and F _(1, 18)_ = 8.25 to 32.37 for flexor muscles, p < 0.01) except the BF muscle (F _(1, 18)_ = 2.71, p = 0.12) since the muscles were activated earlier for translational perturbation than for rotational perturbation. A significant difference between forward/toe up and backward/toe down in the effect on onset latency in the T EXT (F_(1, 18)_ = 12.73), PEC (F_(1, 18)_ = 25.37), L EXT (F_(1, 18)_ = 18.43), and BF muscles (F_(1, 18)_ = 13.03) was demonstrated (p < 0.01), since the T EXT, L EXT, and BF latencies were shorter for forward/toe up perturbation, and the PEC latency was significantly shorter for backward/toe down perturbation. An interaction effect was noted in the ABD R, MG, and TA muscles (p < 0.01). The ABD R (F_(1, 18)_ = 37.80) and MG (F_(1, 18)_ = 55.97) muscles had shorter latency for BT and UR perturbation, while the TA (F_(1, 18)_ = 18.56) muscle had shorter latency for FT and DR perturbation. There was no significant effect of type and direction on the magnitude of muscle activation in any of the muscles (p > 0.01).

### Joint Kinematics

Most of the angular excursions of the head, trunk and lower limbs occurred in the sagittal plane, and postural responses demonstrated a symmetrical pattern. Therefore, the measurements of the postural responses focused on the movement in the sagittal plane, and the angular changes of the lower limb were focused on the right side.

**Figure 3:**
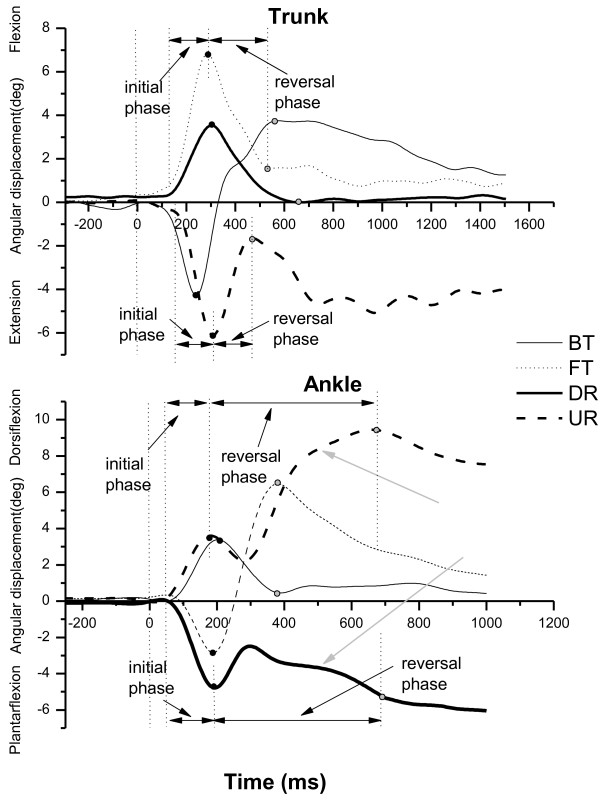
**Angular excursion of the trunk and ankle in four types of perturbation.** Representative trials of the angular excursion of the trunk (top) and the ankle (bottom) during four types of perturbation. A positive value indicates flexion or dorsiflexion, and a negative value indicates extension or plantarflexion. The black dots indicate the initial peak or the first reversal of the angular displacement tracing, and the gray dots indicate the second reversal or the returned baseline. The vertical dotted lines from left to right indicate the onset of the platform movement, the initial joint movements and the reversal movement as well as the end of the reversal movements (second reversal or the returned baseline). The initial movement phase begins from the onset of the initial movement to the onset of the reversal movement. The reversal movement phase begins from the onset of the reversal movement to the end of the reversal movement. The gray arrows indicate the main ankle movement in the reversal movement phase in DR and UR.

The joints all displayed varied degrees of angular change after perturbation, and then the reversal movement was elicited to recover balance in the opposite direction, except at the ankle for rotational perturbations. As examples, Figure 3 shows plots of angular excursion of the trunk and the ankle for four types of perturbation. During DR and UR, initial ankle angular displacement was succeeded by a few degrees of rebound movement and then followed by a second reversal movement that occurred in the same direction as the initial ankle movements. Then, the ankle joint moved to plantar flexion (or dorsiflexion) and remained in that position during DR (or UR).

**Figure 4:**
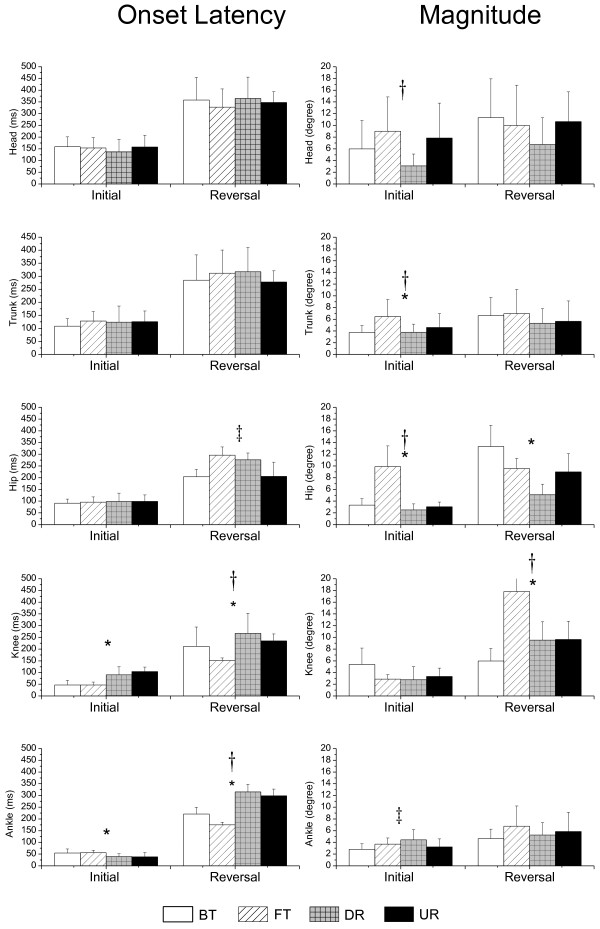
**Group means and standard deviation of the angular displacement.** Group means and standard deviation of onset latency (left) and magnitude (right) of initial and reversal angular changes at the head, trunk, hip, knee and ankle for four types of perturbation. A significant difference between translation and rotation is indicated by *, a significant difference between forward/toe up and backward/toe down is indicated by†, and a significant interaction is indicated by‡.

The group means and standard deviations of onset latency and the magnitude of the initial and reversal angular changes within each of the joints for the four types of perturbation are presented in Figure 4. In terms of the type of perturbation for the initial movement phase, a significant difference in the effect on onset latency was revealed at the distal lower limb, where rotational perturbation induced an earlier initial angular change at the ankle (F_(1, 18)_ = 31.46, p < 0.01) and translational perturbation induced an earlier initial angular change at the knee (F_(1, 18)_ = 122.96, p < 0.01). However, in terms of both type and direction, significant differences occurred in the effect on the magnitude on the proximal upper body, where translation and forward/ toe up direction induced larger changes in the trunk angle (F_(1, 18)_ = 10.13, p < 0.01, F_(1, 18)_ = 10.00, p < 0.01) and hip (F_(1, 18)_ = 81.02, p < 0.01, F_(1, 18)_ = 55.54, p < 0.01), and the forward/toe up direction induced a larger change in the initial head angle (F_(1, 18)_ = 11.42, p < 0.01). There was an interaction effect at the ankle joint (F_(1, 18)_ = 11.12, p < 0.01), and FT and DR induced larger initial ankle angle changes than BT and UR.

For the reversal movement phase, in terms of type and direction, a significant difference in the effect on latency also occurred at the lower limb. Earlier knee flexion (F_(1, 18)_ = 14.62, p < 0.01, F_(1, 18)_ = 29.53, p < 0.01) and ankle dorsiflexion (F_(1, 18)_ = 342.80, p < 0.01, F_(1, 18)_ = 48.47, p < 0.01) were elicited for perturbations in translational and forward/toe up direction. An interaction effect was noted at the hip joint in that earlier hip flexion was elicited for BT and UR (F_(1,18)_ = 66.54, p < 0.01). In terms of type and direction, a significant difference in the effects on magnitude of the change in reversal angle occurred at the hip and knee. A larger change in the hip angle (F_(1, 18)_ = 63.98, p < 0.01) was elicited for translational perturbation, especially in the backward condition (BT). Larger correcting knee movement was elicited for perturbations in translational (F_(1, 18)_ = 11.14, p < 0.01) and forward/toe up direction (F_(1, 18)_ = 85.24, p < 0.01), especially in the combined condition (FT).

### COM displacements

**Figure 5:**
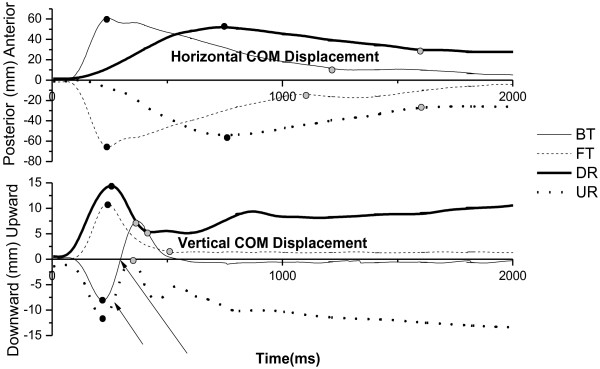
**Horizontal and vertical COM displacements in four types of perturbation.** Representative responses of the horizontal trajectory (top) and the vertical trajectory (bottom) of the body’s COM during four types of perturbation. A positive value indicates that the COM displaces toward the anterior or upward direction, while a negative value indicates that the COM displaces toward the posterior or downward direction. The black dots indicate the initial peak or the first reversal of the COM displacement tracing, and the gray dots indicate the second reversal or the returned baseline. The arrows in the bottom figure indicate the reversal vertical COM displacements in BT and UR.

The means of the maximal medial-lateral COM displacements were less than 10 mm, and there was no significant difference in the effect based on type and direction on the medial-lateral COM displacement (p > 0.05). As expected, the COM moved forward during the backward/toe down direction (BT and DR) and backward during the forward/toe up direction (FT and UR). For vertical displacement, the COM moved upward during FT and DR and downward during BT and UR (Figure 5).

**Figure 6:**
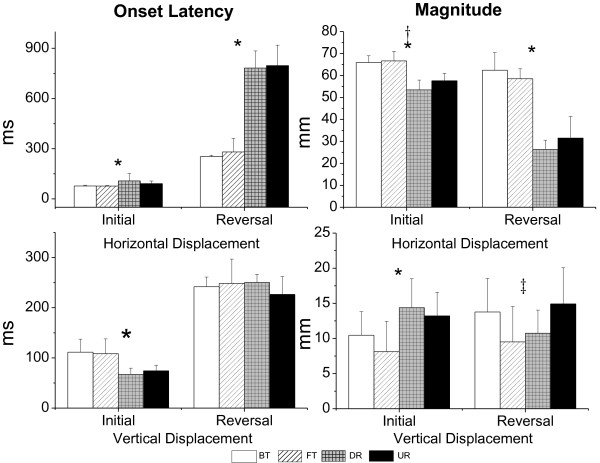
**Group means and standard deviation of COM displacements in the four types of perturbation.** Group means and standard deviation of onset latency (left) and magnitude (right) of horizontal (top) and vertical (bottom) COM displacements for the four types of perturbation. A significant difference between translation and rotation is indicated by *, a significant difference between forward/toe up and backward/toe down is indicated by†, and a significant interaction is indicated by‡.

The means and standard deviations of onset latency and the magnitude of the horizontal (anterior/posterior) and vertical (upward/downward) displacements for the four types of perturbation are presented in Figure 6. For the initial movement phase, in terms of type, significant differences in the effect on onset latency and magnitude were demonstrated on both horizontal and vertical displacements. Translational perturbation induced faster (F_(1, 18)_ = 19.31, p < 0.01) and larger (F_(1, 18)_ = 147.84, p < 0.01) initial horizontal COM displacement, while rotational perturbation induced faster (F_(1, 18)_ = 65.70, p < 0.01) and larger (F_(1, 18)_ = 24.14, p < 0.01) initial vertical displacement. A significant difference in the effect of direction was also demonstrated on initial horizontal COM displacement in that the forward/toe up direction (F_(1, 18)_ = 13.60, p < 0.01) induced larger displacement.

For the reversal movement phase, based on type, a significant difference in the effect on latency and magnitude was on horizontal COM displacement. Translational perturbation corrected faster (F_(1, 18)_ = 483.85, p < 0.01) and recovered more horizontal displacement (F_(1, 18)_ = 706.35, p < 0.01) than rotational perturbation. An interaction effect was noted in reversal vertical COM displacement, where BT and UR induced larger vertical displacement (F_(1, 18)_ = 16.28, p < 0.01).

## Discussion

In this study we examined the effect of the type and direction on onset latency and magnitude of muscle activations, angular changes, and COM displacements. Different types and directions of perturbations have a significant effect on onset latency instead of magnitude of muscle activation. Shorter latency of muscle activation in almost all the muscles was exhibited for translational perturbation, in keeping with expectations. In addition, forward/toe up direction elicited earlier muscle activation in many more muscles, especially in the extensor muscles. We assumed that platform translation and forward/toe up perturbation were more unstable than rotation and backward/toe down, so the postural muscles must activate earlier to recover balance. The PEC muscle activated earlier for backward/toe down. While the PEC muscle is not a pure postural muscle, it flexes, adducts, and rotates the arm medially. For the backward/toe down condition, anterior body sway was induced, and the primary postural response for balance was to move the COM backward. However, since flexing the arm should move the COM forward and result in further instability, the earlier arm movement did not act to counterbalance the movement of the COM but acted as a natural defense, presumably in an initial attempt to cushion the impact of an impending fall [31].

Two phases of joint movements and COM displacements were quantified to obtain kinematic data. The onset latency of the initial movement in the lower extremity, ranging from 39 to99 ms, was less than the muscle activation latency. These initial movements should present passive responses to the perturbations resulting from the immediate biomechanical consequence of the platform movement. In our study, ramp onset and ramp offset acceleration/deceleration profiles were set with a short acceleration-deceleration interval. For very brief ramp displacement (e.g., 140 ms), the platform deceleration ended before the initial response (i.e., the acceleration response). The reversal movement may present active movement, although the platform braking effect may influence joint movement [32,33]. The results showed that the onset latency of the reversal movement was usually longer than the time required for muscle activation. In addition, the difference between the onset of muscle responses and the onset of the reversal movement was small, and the direction of the reversal movement was opposite to the initial movement [18]. Therefore, the onset latency and the magnitude of the reversal movement were measured as the onset and magnitude of the active balance reaction.

The results of this study demonstrated that balance disturbances induced by platform movement and the balance reaction controlled by the CNS were all influenced by the type or direction of the support surface perturbation. The significant difference of initial effect on onset latency was demonstrated on the ankle and knee joints, while the significant difference of the initial effect on the magnitude was on the upper body. This occurred because the distal joints, which were closer to the platform, were influenced quickly by the perturbations, whereas the initial movement caused by the inertia effect of the platform acceleration was enhanced by the large body mass. The proximal upper body, which has a larger body mass, would lead to a greater change in the angle. Rotational perturbations rotated the ankle joint, directly inducing earlier initial ankle movement, while translational perturbation, which was imposed on the lower leg and produced much more horizontal force, induced earlier initial knee movement. Translational and forward/toe up perturbation, which induces posterior falling of the body’s COM, appeared to impose larger changes in head and trunk angles, resulting in greater upper body instability. Thus, proprioceptive input at the ankle joint was the primary stimulus provided by rotational perturbation, whereas vestibular stimulus as a result of head movement and proprioceptive input in other than the ankle joint can be provided by translational and forward/toe up perturbation.

An interaction effect on the magnitude of the initial angular change in the ankle showed that FT and DR induced larger initial ankle angular changes than BT and UR. Presumably, this occurred during BT and UR because the ankle joint was rotated upward and the gastrocnemius was stretched. When standing with the knee in full extension, tension in the gastrocnemius restricts the range of dorsiflexion at the ankle. Although the TA muscle was also stretched by plantar flexion, a larger angular change was induced by DR and FT because the TA muscle is a one-joint muscle, in contrast to the gastrocnemius, a two-joint muscle, that underwent different changes in length with similar stretch.

In the reversal movement phase, the results of joint kinematics were similar to the EMG result; some joint movements were elicited earlier for translational and forward/toe up perturbation. An interaction effect on onset latency was also demonstrated. Hip flexion had shorter latency for BT and UR perturbation. In contrast to the EMG results, in which there were no significant effects on magnitude, there was a significant effect on magnitude in joints. Larger hip flexion was elicited for BT, and larger knee flexion was elicited for FT. A previous study reported that hip strategy is a highly effective way to stabilize upright posture [34], but, in our study, the participant was tested while standing upright with hip-knee extension, so the available range of hip extension was limited. For FT, it is reasonable to assume that knee flexion accompanied by hip extension will contribute to bringing the COM forward to compensate for the limited hip extension. Prior studies have suggested that trunk movements provide an important trigger for postural responses [35,36] and that trunk stabilization is the major task for the CNS to control balance [37]. Although the larger imposed trunk movement occurred in translational and forward/toe up perturbation in our study, no significant effect of types or directions was noted in reversal trunk movement because the postural balance was achieved through a combination of trunk, hip, and lower leg movements rather than by the trunk alone.

Postural stability is restored by multi-segmental movement synergies that require inter-joint coordination. However, joint movement that occurs in the absence of muscle activities could result from inter-segmental mechanical effects of internal and weight forces arising from the movement of adjacent joints or from several muscle synergies that vary with time and perturbation direction [38]. Most of the joint movements were not caused by only one muscle, and the muscle may act as concentric or eccentric contraction. Therefore, even in the larger hip or knee angular changes elicited for BT or FT, the effect of type or direction on magnitude didn’t manifest on muscles in our findings, although a previous study demonstrated that the magnitude of muscle activities varied as a function of platform acceleration/velocity [18]. Furthermore, the velocity of the movement of the platform was similar for the four perturbations in our study, which explains why there was no significant effect on the magnitude of muscle activity.

Rotational perturbations stretched the same leg muscles as translational perturbations, while eliciting a different pattern of reversal ankle movements. Translational perturbations elicited stretch reflexes and balance-correcting responses in the same ankle muscles [39]. That was the agonist muscle reaction, in which the reversal ankle movement was in the opposite direction to the initial movement. During rotational perturbations, the main ankle movement in the reversal phase was in the same direction as the imposed initial ankle movement (Figure 3). This finding corresponded with the description of the long-latency antagonist reaction in the ankle muscle in response to postural imbalance in Nardone’s study [14].

The results of this study provided evidence to support the view that translational perturbations induce more horizontal COM displacements than rotational perturbations and that rotational perturbations induce larger vertical COM displacements [12]. In the initial movement phase, platform rotations also caused the COM to shift backward or forward, while horizontal displacement did so to a lesser degree and with longer onset latency because the rotational perturbation did not move the support surface substantially away from the position of the COM, although there also was a linear translation produced by platform rotation. Mechanically, during translational perturbations, the platform moved 70 mm, which would shift the COM with respect to the support surface equal with the platform’s 70 mm displacement, whereas during rotational perturbations, a platform rotation of seven degrees only resulted in about a 12.2 mm (100 mm × sin7°) translation with a 12.1 mm (12.2 mm × cos7°) horizontal component and a 1.5 mm (12.2 mm × sin7°) vertical component. In joint kinematics, the forward/toe up direction induced larger changes in the head, trunk, and hip angles. The trunk and hip angular displacements had an in-phase pattern in response to the perturbation. Undoubtedly, these larger angular changes would lead to a larger horizontal displacement of the COM during forward/toe up perturbation. In the reversal movement phase, larger and faster horizontal COM displacements also were elicited by translational perturbations. The low and incomplete recovery of the COM displacement was due to the platform’s remaining in a rotational up or down position after the rotational movement and by changing the end COM positions by bringing the COM forward in DR and backward in UR (Figure 5). For vertical COM displacement, larger reversal displacement was elicited for UR and BT (instead of DR); as the vertical trajectories of the COM displacement shown in Figure 5 make clear, there were rebound (first reversal) and return (second reversal) responses. We assume that BT and UR rotated the ankle joint and stretched the gastrocnemius, inducing a reflex-based plantar flexion, which raised the heels up and produced larger vertical COM displacement in the reversal movement phase.

### Study limitations

In this study, only the right leg was tested without considering the leg dominance. Symmetrical behavior in the lower limbs has been assumed for simplicity. However, lower limb asymmetry has been noted throughout the literature [40,41]. Further studies may focus on testing performance of the dominant leg to reveal the effects of perturbation on postural responses. In our study, we use the linear envelope method for analysis of EMG signals because it was a simple and widely used method in human movement research. However, we acknowledge that there are other advanced signal processing methods including wavelet method, double threshold algorithm and Generalized Likelihood Ratio test [42-44], that can provide more accurate and precision information. These state-of-the-art methods offer a potentially fruitful line of data processing and may be considered for future research.

## Conclusion

In summary, the findings of this study support the supposition that the translational and forward/toe up perturbations induce larger upper body movements and faster and larger horizontal displacement of the COM, which lead to increased instability. For balance reaction, hip flexion accompanied by arm movement, which acts as a natural defense, was elicited for backward translation, and hip extension combined with knee flexion was elicited for forward translation. Recovery balance from a perturbed surface is one important control strategy for postural control. Older adults and persons with neurologic deficit may change the behavior of postural responses or impair movement strategies during perturbed stance. The perturbation-based assessment system or training programs are commonly developed in clinical or laboratory setting for assessing and treating postural disorders. For assessment, different types of perturbation pose different level of instability that influences postural strategies. For balance training, the amplitude, velocity and direction of perturbations can be varied to practice in-place or stepping reaction and normal strategy used to respond to an external perturbation can be taught to the patients. The results of this study provided some insights for selecting appropriate support surface perturbations for assessment and for designing methods for training postural control.

## Abbreviations

COM: Center of mass; BOS: Base of support; EMG: Electromyography; FT: Forward translation; BT: Backward translation; UR: Toe-up rotation; DR: Toe-down rotation; NK EXT: cervical paraspinae; NK FLX: Neck flexor; T EXT: Thoracic paraspinae; PEC: Pectoralis major; L EXT: Lumbar paraspinae; ABD R: Abdominal rectus; BF: Biceps femoris; RF: Rectus femoris; MG: Medial gastrocnemius; TA: Tibialis anterior; GCVSPL: Generalized cross-validation spline smoothing; ISB: International Society of Biomechanics; ANOVA: Analyses of variance.

## Competing interest

The authors declare that they have no competing interests.

## Authors’ contributions

CLC contributed to conception, experimental design, data interpretation, and write the manuscript. SZL and HWW contributed to data analysis and interpretation. SKW contributed to data collection and helped to interpret the results. KTY aided in data acquisition and subject recruitment. FCS contributed to conception and critically revised the manuscript. All authors read and approved the final manuscript.
